# Serine, *N*-acetylaspartate differentiate adolescents with juvenile idiopathic arthritis compared with healthy controls: a metabolomics cross-sectional study

**DOI:** 10.1186/s12969-022-00672-z

**Published:** 2022-02-10

**Authors:** Kimberly A. Lewis, Nico Osier, Ruy Carrasco, Jennifer Chiou, Patricia Carter, Alexandra Garcia, Elena Flowers, Efstathios D. Gennatas, Christina Nguyen, Ambreen Rana, Sharon A. Brown, Stefano Tiziani

**Affiliations:** 1 Department of Physiological Nursing, School of Nursing, University of California, San Francisco, 2 Koret Way, San Francisco, CA 94131 USA; 2 School of Nursing, The University of Texas at Austin, 1710 Red River St, Austin, TX 78712 USA; 3 Department of Neurology, Dell Medical School, Austin, TX 78701 USA; 4 Texas A&M University, 400 Bizzell St., College Station, TX 77843 USA; 5 Department of Nutritional Sciences, College of Natural Sciences, The University of Texas at Austin, Austin, TX USA; 6 Capstone College of Nursing, University of Alabama, 650 University Blvd E, Tuscaloosa, AL 35401 USA; 7 Family and Public Health Nursing and Nursing Administration Departments, School of Nursing, The University of Texas at Austin, 1710 Red River St, Austin, TX 78712 USA; 8 Department of Epidemiology and Biostatistics, School of Medicine, University of California at San Francisco, 513 Parnassus Ave, San Francisco, CA 94117 USA; 9 Nursing Research, Dell Children’s Medical Center of Central Texas, 4900 Mueller Blvd, Austin, TX 78723 USA; 10 Department of Pediatrics, Dell Medical School, The University of Texas at Austin, Austin, TX USA

**Keywords:** Juvenile idiopathic arthritis, Metabolomics, Adolescents, *N*-acetylaspartate, Serine, Biomarkers, Precision medicine, Systems biology

## Abstract

**Background:**

In comparison with the general population, adolescents with juvenile idiopathic arthritis (JIA) are at higher risk for morbidity and mortality. However, limited evidence is available about this condition’s underlying metabolic profile in adolescents with JIA relative to healthy controls. In this untargeted, cross-sectional metabolomics study, we explore the plasma metabolites in this population.

**Methods:**

A sample of 20 adolescents with JIA and 20 controls aged 13–17 years were recruited to complete surveys, provide medical histories and biospecimens, and undergo assessments. Fasting morning plasma samples were processed with liquid chromatography–mass spectrometry. Data were centered, scaled, and analyzed using generalized linear models accounting for age, sex, and medications (*p*-values adjusted for multiple comparisons using the Holm method). Spearman’s correlations were used to evaluate relationships among metabolites, time since diagnosis, and disease severity.

**Results:**

Of 72 metabolites identified in the samples, 55 were common to both groups. After adjustments, 6 metabolites remained significantly different between groups. Alpha-glucose, alpha-ketoglutarate, serine, and *N*-acetylaspartate were significantly lower in the JIA group than in controls; glycine and cystine were higher. Seven additional metabolites were detected only in the JIA group; 10 additional metabolites were detected only in the control group. Metabolites were unrelated to disease severity or time since diagnosis.

**Conclusions:**

The metabolic signature of adolescents with JIA relative to controls reflects a disruption in oxidative stress; neurological health; and amino acid, caffeine, and energy metabolism pathways. Serine and *N*-acetylaspartate were promising potential biomarkers, and their metabolic pathways are linked to both JIA and cardiovascular disease risk. The pathways may be a source of new diagnostic, treatment, or prevention options. This study’s findings contribute new knowledge for systems biology and precision health approaches to JIA research. Further research is warranted to confirm these findings in a larger sample.

**Supplementary Information:**

The online version contains supplementary material available at 10.1186/s12969-022-00672-z.

## Background

Juvenile idiopathic arthritis (JIA) is defined as persistent joint swelling (≥6 weeks) often accompanied by pain, heat, limited range of motion, erythema, or loss of use, with an onset prior to age 16 years [1–3]. It is the most common rheumatic disease in adolescents [1]. Although JIA’s exact etiology is unknown, it is thought to arise in genetically susceptible individuals who are influenced by environmental factors [1–3]. Across regions internationally, JIA’s incidence ranges from 1 to 22 per 100,000 children under age 18 years, and its prevalence ranges from 7 to 400 per 100,000 children [1, 4]. Among adolescents with all types of JIA, morbidity and mortality are higher than in the general population [5–8]. As a result, there is a need for a better understanding of JIA pathogenesis to improve prevention, diagnosis, and treatment options.

Metabolomics offers a strategy to study the metabolic dysfunction that precedes clinically detectable disease onset [9–11]. Untargeted metabolomics is the comprehensive study of metabolic byproducts present in bodily fluids or tissues, usually in comparison with a control group [10]. In preclinical and clinical studies of rheumatoid arthritis, metabolomics analysis of serum, plasma, urine, and synovial fluid has identified diagnostic biomarkers and indicators of treatment responsiveness [10, 12], but this is only beginning to be explored in adolescents with JIA. The purpose of the present untargeted metabolomics study is to describe the metabolic profile in the plasma of adolescents aged 13–17 years with JIA in comparison with a control group of healthy adolescents.

## Methods

This was a cross-sectional study to compare adolescents aged 13–17 years with JIA (*n* = 20) and age, sex, race, ethnicity, and body mass index (BMI) percentile matched healthy controls (*n* = 20). Participants were recruited from the greater Austin, Texas metropolitan area, which comprises over 1.8 million people. Central Texas’s racial and ethnic demographic composition is 48.9% non-Hispanic White; 33.6% Hispanic; 8.9% Black; and 8.7% Pacific Islander, Asian, or American Indian [13]. JIA participants were recruited through pediatric rheumatologist referrals, emailed flyers, in person, and social media. Control group participants were recruited via flyer distribution throughout the community and referral from other participants. The study received ethics approval from The University of Texas at Austin Institutional Review Board.

Participants were enrolled from August 2018 to October 2019. Adolescents were included in the JIA group if they had a prior clinical diagnosis of JIA from the patient’s pediatric rheumatologist and were able to complete surveys in English or Spanish. Participants in the control group were healthy and without a rheumatologic disease diagnosis. Participants were excluded from either group if they were pregnant via self-report and confirmed with urine tests after biospecimen collection. Study visits took place at the pediatric rheumatology clinic and were scheduled to begin in the morning when participants had fasted for at least 8 h to standardize the time of day for biospecimen collection. Participants first completed surveys, and the study team then collected anthropometric measures (height, weight), blood pressure, and biospecimens (blood and urine).

Data collected included demographics, JIA disease activity and history (JIA subtype, rheumatoid factor, physician’s global assessment of disease activity [14, 15], time since diagnosis, age at diagnosis, and a pain visual analog scale from 0 to 10) [16]; medications taken within the 7 days prior to the study visit, and BMI percentile. Blood was collected by venipuncture by experienced pediatric phlebotomists or nurses using a standardized protocol. To account for potential confounding effects of dietary intake, detailed dietary data were collected via a 3-day food diary for the 3 days prior to the study visit and were analyzed using 2019 Nutrition Data System for Research software [17, 18].

### Plasma sample preparation and metabolomics analysis

Plasma was separated from whole blood using centrifugation, then the plasma was immediately flash frozen using liquid nitrogen and stored at − 80 °C to halt metabolism [19, 20]. After completion of recruitment and patient sample collection, frozen plasma was thawed on ice and filtered by ultracentrifugation in Nanosep 3 K Omega Membrane filters (Pall Corporation, Port Washington, NY) at 4 °C [20].

The plasma filtrate was diluted in high-performance liquid chromatography grade water at a ratio of 1:500 and transferred to liquid chromatography–mass spectrometry vials for analysis. Samples were spiked at a ratio of 1:10 with deuterated internal standards to track system performance [21–23]. Briefly, polar metabolite analysis was conducted on a Vanquish Flex ultra-performance liquid chromatography system (Fisher Scientific, San Jose, CA). Mobile phases of (A) water and 0.2% formic acid and (B) methanol were used with a Kinetex 2.6 μm C18 100 Å, 150 × 2.1 mm high-performance liquid chromatography column (Phenomenex, Torrance, CA) at a flow rate of 150 μL/min at an A/B ratio of 98/2 for 4 min, 20/80 for 10 min, 2/98 for 1 min, held at this ratio for 6 min, and finally 98/2 for 14 min. Sample injection volume was 5 μL. In tandem with the Vanquish, the Q Exactive Hybrid Quadrupole Orbitrap mass spectrometer (Thermo Scientific, Bremen, Germany), equipped with electrospray ionization, was used to acquire untargeted polar metabolite data in positive/negative ion switching mode. Pooled quality control samples were acquired every 6 samples and were used to monitor instrument stability for background subtraction. Acquisition parameters were set as follows: spray voltage, 3.5 kV; capillary temperature, 320 °C; sheath gas, 45 (arbitrary units); auxiliary gas, 10 (arbitrary units); m/z range, 70–1000 (HILIC), 50–750 (RP); data acquisition, centroid mode; microscans, 10; AGC target, 1e6; maximum injection time, 200 milliseconds; mass resolution, 70,000 FWHM at m/z 200.

Raw data were imported into SIEVE 2.2.0 SP2 software (Thermo Scientific, San Jose, CA) to conduct peak picking and spectral alignment. Integrated peak area, mass to charge ratio, and retention time were exported from SIEVE. Metabolite identification from the SIEVE export was achieved by matching mass to charge ratio and retention time to a library of compounds using an in-house MATLAB script (IROA 300, Mass Spectrometry Metabolite Library of Standards; IROA Technologies, Sea Girt, NJ). Peaks were excluded from further analysis if the coefficient of variance exceeded 0.25 as calculated by integrated peak areas of repeat injects of a pooled quality control. Probabilistic quotient normalization was conducted to normalize integrated peak area [24].

### Data analysis

To test for differences in metabolite levels between JIA cases and controls, the data were centered, scaled, and fit using linear regression in R v4.1.0 [25]. The R code is available in Supplementary File 1. Significance levels (*p*-values) were adjusted for multiple comparisons using the Holm method [26]. Models were fit as follows for each metabolite:

$$metabolit{e}_i\sim {\beta}_0+{\beta}_1 Group+{\beta}_2 Age+{\beta}_3 Sex+{\beta}_4 MedsRheum+{\beta}_5 MedsNonrheum,$$

where *β*_0_ is the intercept term and *β*_1_to *β*_5_ are the linear coefficients.

Correlation coefficients between metabolites and disease activity, as well as between metabolites and time since diagnosis, were estimated using Spearman’s rho, and associated *p*-values were also adjusted for multiple comparisons using the Holm method [27]. Although we were prepared to train multivariable models using partial least squares discriminant analysis, this was unnecessary due to mass univariate testing results. Metaboanalyst 5.0 enrichment analysis was used to evaluate the top 25 Kyoto Encyclopedia of Genes and Genomes metabolic pathways implicated in the results [28, 29]. Differences between groups for sample characteristics were evaluated using chi square and two-tailed *t*-tests for independent samples. A sensitivity analysis was run to identify any metabolites significantly different between RF positive and RF negative JIA as a potential confounder. Since there were no previously available studies from which to determine effect sizes, the sample size was based on the aim of providing initial evidence to be confirmed with future research.

## Results

Forty participants (*n* = 20 with JIA; *n* = 20 controls) were enrolled in the study. However, one JIA participant’s data was excluded prior to data analysis due to a previously unidentified comorbidity. Thus, the final sample was *N* = 39 participants. Participants’ characteristics are detailed in Table 1. The overall sample ranged in age from 13 to 17 years, with a mean age of 14.6 ± 1.5. Self-reported demographics indicate that the sample was 69% female, 33% Hispanic or Latino ethnicity, 8% Black, and 92% White. We were unable to detect a statistically significant difference between the JIA and control groups’ demographics (*p* < 0.05) for age, sex, race, or ethnicity. Dietary micro- and macronutrient intake did not differ between the JIA group and controls.

**Table 1:** Demographics and Clinical Characteristics of Study Participants by Group: Adolescents Aged 13–17 Years with Juvenile Idiopathic Arthritis versus Healthy Controls

Descriptor	JIA Group (***n*** = 19)		Control Group (***n*** = 20)		χ^**2**^	***p***
	***n***	%	***n***	%		
**Sex**						
Male	6	32	6	30	0.011	0.915
Female	13	68	14	70		
**Race**						
Black	1	5	2	10	0.308	0.579
White	18	95	18	90		
**Ethnicity**						
Hispanic/Latino	6	32	7	35	0.051	0.821
Not Hispanic/Latino	13	68	13	65		
	***Mean***	***SD***	***Mean***	***SD***	***t***	***p***
Age (years)	15.0	1.4	14.3	1.4	1.636	0.110
BMI percentile	57.5	28.2	57.5	27.1	0.003	0.998
Pain Visual Analog Scale	2.1	2.1	0.5	1.0	−3.229	0.003*

*Significant at the *p* < 0.05 level

### JIA disease history

The JIA group’s mean age at diagnosis was 7.5 ± 5.2 years, with mean time since diagnosis at 7.5 ± 5.1 years. Their mean pain score was 2 ± 2 on the visual analog scale from 0 (*no pain*) to 10 (*worst pain*) [16]. JIA subtypes were 42.1% idiopathic, 26.3% polyarticular, 21.1% undifferentiated, 5.3% psoriatic, and 5.3% systemic. Two patients were rheumatoid factor positive and had the highest rating on the physician’s global assessment of disease activity scale [14, 15], with a mean score of 9 out of 10. Eight were in inactive disease based on a disease activity score of ≤1 and clinical evaluation by the pediatric rheumatologist [29, 30]. Eleven were in active disease at the time of the study visit; their mean score was 5.1 ± 3.1. Most of the sample fell within the normal body weight percentile for height, age, and sex. Fifteen percent of those in the control group and 21% of the JIA group were overweight or obese (BMI percentile > 85).

### Medications, vitamins, and supplements

Adolescents in both groups reported taking medications within the past 7 days (Supplementary Table 1). Our generalized linear model analysis controlled for medications as a covariate. Because the count in each category of medications was small, and because adolescents in both groups reported taking both immunomodulators and non-immunomodulators, medications were dichotomized and coded as either immunosuppressants/modulators (i.e., biologic and nonbiologic disease-modifying anti-rheumatic drugs, corticosteroids, and nonsteroidal anti-inflammatory drugs) or non-immunosuppressants/modulators (i.e., vitamins, hormones, or seasonal allergy medications).

### Metabolites

A total of 72 metabolites were identified in the samples. Sixty-five metabolites were identified in the control group and 62 in the JIA group (Table 3). Fifty-five of the identified metabolites were common to both groups. Ten metabolites were found in the control group that were not detected in the JIA group samples: 2-hydroxypyridine, 3-aminoisobutanoate, 3-ureidopropionate, 5-aminolevulinic acid, isocitric acid, glutamic acid, lysine, acetyl-alanine, pyridoxal, taurine. Seven metabolites were unique to the JIA group and undetected in the control group: theobromine, acetyl-lysine, *N*-acetyl-phenylalanine, ornithine, glutarate, cytidine monophosphate (CMP), and methylglutaric acid. No metabolites were significantly related to disease activity or time since diagnosis.

**Fig. 1:**
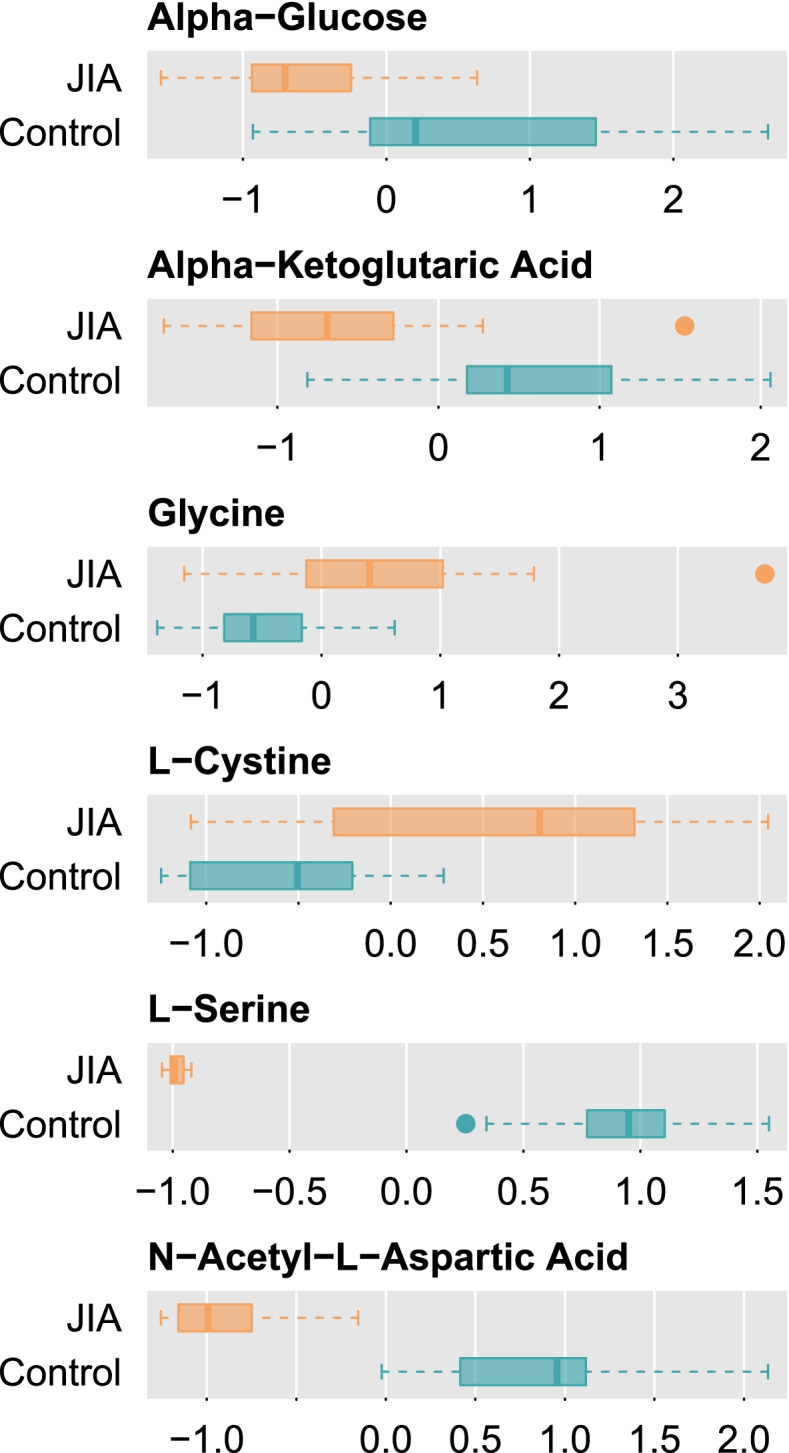
Box Plots of Metabolites Significantly Different Between JIA and Control Groups (*p* < 0.05). Metabolites were centered, scaled, and standardized prior to analysis

After controlling for age, sex, and medications, 6 of the metabolites present in samples from both groups were significantly different (Holm-adjusted *p* < 0.05) between groups: alpha-glucose, alpha-ketoglutarate, glycine, cystine, serine, and *N*-acetylaspartate (NAA). The latter two showed perfect separation between the JIA and control groups (Fig. 1, Table 2). The total separation between groups in serine and NAA obviated the use of multivariate dimensionality reduction analysis or a multivariable model. RF was not found to be a confounder after the sensitivity analysis.

**Table 2:** Overview of the Metabolites Identified as Significantly Different (adjusted *p* < 0.05) in Adolescents with Juvenile Arthritis Relative to Controls

Metabolite	Category^a^	Integrated Peak Area for JIA Group Relative to Control Group
Alpha-glucose	Hexose—a monosaccharide in which the sugar unit is a six-carbon containing moiety.	↓
Alpha-ketoglutarate (a.k.a. oxoglutaric acid, alpha-ketoglutarate)	Gamma keto acids and derivatives, integral part of the tricarboxylic acid (TCA) cycle, a key part of aerobic metabolism.	↓
Glycine	Nonessential amino acid involved in the body’s production of DNA, phospholipids, and collagen, and in the release of energy.	↑
Cystine	Formed from two cysteine molecules joined together, found in hair, skin, and nails; cysteine is a sulfur-containing alpha amino acid found in most proteins and contains a thiol group.	↑
Serine	Conditionally essential amino acid derived from glycine.	↓
*N*-acetylaspartate	Derivative of aspartic acid, a nonessential amino acid made from glutamic acid; may provide resistance to fatigue and promote endurance.	↓

^a^From Wishart DS, Feunang YD, Marcu A, Guo AC, Liang K, Vásquez-Fresno R, et al. HMDB 4.0: the human metabolome database for 2018. Nucleic Acids Res. 2018;46(D1):D608–17

**Table 3:** Integrated Peak Areas of Metabolites Identified from Blood Samples in Adolescents with Juvenile Arthritis vs Controls

Metabolite	JIA		Control		∆ (JIA Relative to Control)	
	***Mean***	***SD***	***Mean***	***SD***	Mean Difference	% Difference
2-hydroxypyridine	.	.	3.98e+ 5	2.13e+ 5	−3.98e+ 05	**
3-aminoisobutanoate	.	.	7.05e+ 7	1.81e+ 7	−7.05e+ 07	**
3-hydroxykynurenine	6.09e+ 5	2.41e+ 5	6.86e+ 5	4.14e+ 5	− 7.70e+ 04	−11%
3-methylglutaric acid	3.57e+ 6	9.5e+ 5	.	.	+ 3.57e+ 06	**
3-ureidopropionate	.	.	2.77e+ 6	5.71e+ 5	−2.77e+ 06	**
4-guanidinobutanoate	1e+ 6	5.61e+ 5	9.98e+ 5	8.23e+ 5	+ 2.00e+ 03	0%
5, 6-dihydrouracil	1.11e+ 7	1.76e+ 6	1.12e+ 7	1.47e+ 6	−1.00e+ 05	− 1%
5-aminolevulinic acid	.	.	1.87e+ 7	6.05e+ 6	−1.87e+ 07	**
5-methylcytosine	5.95e+ 5	2.05e+ 5	6.38e+ 5	2.19e+ 5	−4.30e+ 04	−7%
5-oxolproline	3.25e+ 8	6.39e+ 7	3.38e+ 8	4.92e+ 7	−1.30e+ 07	− 4%
Acetyl-carnitine	1.13e+ 9	3.66e+ 8	1.31e+ 9	3.3e+ 8	−1.80e+ 08	− 14%
Acetyl-lysine	5.41e+ 5	2.7e+ 5	.	.	+ 5.41e+ 05	**
Adenine	5.18e+ 5	1.77e+ 5	4.99e+ 5	2.81e+ 5	+ 1.90e+ 04	4%
Alpha-glucose	2.93e+ 7	3.85e+ 6	3.71e+ 7	7.42e+ 6	−7.80e+ 06	−21%^±^
Alpha-ketoglutarate	3.38e+ 7	7.64e+ 6	4.63e+ 7	7.85e+ 6	−1.25e+ 07	− 27%^±^
Arginine	1.05e+ 8	2.42e+ 7	1.02e+ 8	1.79e+ 7	+ 3.00e+ 06	3%
Asparagine	1.11e+ 7	3.07e+ 6	1.06e+ 7	3.25e+ 6	+ 5.00e+ 05	5%
Beta-alanine	1.82e+ 8	3.74e+ 7	1.87e+ 8	3.66e+ 7	− 5.00e+ 06	−3%
Betaine	7.47e+ 8	2.29e+ 8	7.93e+ 8	1.97e+ 8	−4.60e+ 07	−6%
Carnitine	1.35e+ 9	2.04e+ 8	1.28e+ 9	1.44e+ 8	+ 7.00e+ 07	5%
Citrate	5.11e+ 8	1.44e+ 8	7.66e+ 8	1.85e+ 8	−2.55e+ 08	−33%
Citrulline	4.7e+ 7	8.54e+ 6	5.43e+ 7	9.93e+ 6	−7.30e+ 06	−13%
CMP	3.81e+ 5	3.12e+ 5	.	.	+ 3.81e+ 05	**
Creatine	4.2e+ 8	1.74e+ 8	4.05e+ 8	1.78e+ 8	+ 1.50e+ 07	4%
Creatinine	3.39e+ 8	5.66e+ 7	3.41e+ 8	5.12e+ 7	−2.00e+ 06	−1%
Cystine	8.58e+ 6	3.66e+ 6	4.51e+ 6	1.65e+ 6	+ 4.07e+ 06	90%^±^
Cytidine	7.85e+ 5	5.72e+ 5	5.91e+ 5	5.59e+ 5	+ 1.94e+ 05	33%
Deoxycarnitine	8.06e+ 7	1.35e+ 7	7.71e+ 7	1.44e+ 7	+ 3.50e+ 06	5%
Fumarate	1.93e+ 6	5.89e+ 5	2.21e+ 6	6.47e+ 5	−2.80e+ 05	−13%
Glutamic acid	.	.	1.36e+ 7	3.98e+ 6	−1.36e+ 07	**
Glutamine	2.88e+ 8	3.8e+ 7	3.06e+ 8	4.28e+ 7	−1.80e+ 07	−6%
Glutarate	3.39e+ 6	2.07e+ 6	.	.	+ 3.39e+ 06	**
Glycerol	2.14e+ 7	1.01e+ 7	2.6e+ 7	9.92e+ 6	−4.60e+ 06	−18%
Glycine	1.73e+ 7	5e+ 6	1.26e+ 7	2.36e+ 6	+ 4.70e+ 06	37%^±^
Guanosine	7.21e+ 6	3.28e+ 6	7.07e+ 6	3.66e+ 6	+ 1.40e+ 05	2%
Histidine	5.09e+ 7	6.42e+ 6	5.64e+ 7	8.73e+ 6	−5.50e+ 06	−10%
Homocysteine	1.96e+ 7	1.42e+ 7	2.52e+ 7	1.71e+ 7	− 5.60e+ 06	−22%
Inosine	4.76e+ 7	2.18e+ 7	4.66e+ 7	2.57e+ 7	+ 1.00e+ 06	2%
Isocitric acid	.	.	1.93e+ 7	3.82e+ 6	−1.93e+ 07	**
Isoleucine	3.51e+ 9	4.35e+ 8	3.63e+ 9	6.72e+ 8	−1.20e+ 08	−3%
Kynurenine	8.73e+ 6	3.14e+ 6	1.06e+ 7	1.23e+ 7	−1.87e+ 06	− 18%
Lactate	1.76e+ 9	4.64e+ 8	2.16e+ 9	6.69e+ 8	−4.00e+ 08	− 19%
Leucine	7.31e+ 9	6.18e+ 8	7.84e+ 9	1.38e+ 9	−5.30e+ 08	−7%
Lysine	.	.	1.54e+ 8	1.7e+ 7	−1.54e+ 08	**
Malate	3.15e+ 7	6.73e+ 6	3.86e+ 7	5.71e+ 6	−7.10e+ 06	−18%
Methionine	1.15e+ 9	1.51e+ 8	1.22e+ 9	1.37e+ 8	−7.00e+ 07	−6%
Methyl-histidine	1.28e+ 7	6.66e+ 6	1.03e+ 7	5.64e+ 6	+ 2.50e+ 06	24%
Myristic acid	2.21e+ 5	1.5e+ 5	3.69e+ 5	1.35e+ 5	−1.48e+ 05	−40%
*N*-acetyl-alanine	.	.	1.17e+ 7	1.1e+ 6	−1.17e+ 07	**
*N*-acetyl-aspartate	1.5e+ 6	2.34e+ 5	3.01e+ 6	4.5e+ 5	−1.51e+ 06	−50%^±^
*N*-acetyl-glycine	1.68e+ 7	8.35e+ 6	1.84e+ 7	6.24e+ 6	−1.60e+ 06	− 9%
*N*-acetyl-methionine	1.82e+ 6	7.04e+ 5	1.75e+ 6	3.67e+ 5	+ 7.00e+ 04	4%
*N*-acetyl-phenylalanine	1.69e+ 5	1.31e+ 5	.	.	+ 1.69e+ 05	**
*N*-acetyl-putrescine	2.11e+ 6	3.79e+ 5	2.33e+ 6	5.17e+ 5	−2.20e+ 05	−9%
*N*-acetyl-serine	3.97e+ 6	5.16e+ 5	4.5e+ 6	6.29e+ 5	−5.30e+ 05	−12%
Ornithine	5.41e+ 7	1.61e+ 7	.	.	+ 5.41e+ 07	**
Pantothenic acid	7.56e+ 6	4.02e+ 6	7.23e+ 6	2.64e+ 6	+ 3.30e+ 05	5%
Phenylalanine	4.68e+ 9	3.38e+ 8	5.01e+ 9	4.1e+ 8	−3.30e+ 08	−7%
Pipecolate	2.97e+ 8	1.77e+ 8	3.73e+ 8	3.56e+ 8	− 7.60e+ 07	− 20%
Proline	1.56e+ 9	3.41e+ 8	1.89e+ 9	5.3e+ 8	− 3.30e+ 08	−17%
Pyridoxal	.	.	8.56e+ 5	3.82e+ 5	−8.56e+ 05	**
Ribose-5-phosphate	7.47e+ 5	2.63e+ 5	7.62e+ 5	2.44e+ 5	−1.50e+ 04	−2%
Serine	2.76e+ 5	1.36e+ 5	7.47e+ 6	1.21e+ 6	−7.19e+ 06	−96%^±^
Spermidine	8.1e+ 6	4.13e+ 6	7.77e+ 6	4.17e+ 6	+ 3.30e+ 05	4%
Succinate	4.36e+ 7	1e+ 7	4.88e+ 7	9.73e+ 6	−5.20e+ 06	−11%
Taurine	.	.	9.82e+ 6	3.41e+ 6	−9.82e+ 06	**
Theobromine	2.04e+ 8	4.93e+ 8	.	.	+ 2.04e+ 08	**
Threonine	8.05e+ 7	1.99e+ 7	8.39e+ 7	1.4e+ 7	−3.40e+ 06	−4%
Trans-aconitate	2.32e+ 6	8.61e+ 5	2.85e+ 6	7.49e+ 5	−5.30e+ 05	−19%
Tyrosine	2.07e+ 9	3.15e+ 8	2.31e+ 9	3.45e+ 8	−2.40e+ 08	− 10%
Uracil	1.49e+ 6	4.72e+ 5	2.03e+ 6	5.37e+ 5	−5.40e+ 05	− 27%
Urocanate	8.31e+ 6	5.09e+ 6	9.99e+ 6	6.89e+ 6	−1.68e+ 06	− 17%

^±^adjusted *p* < 0.05

**detected in one group only

**Fig. 2:**
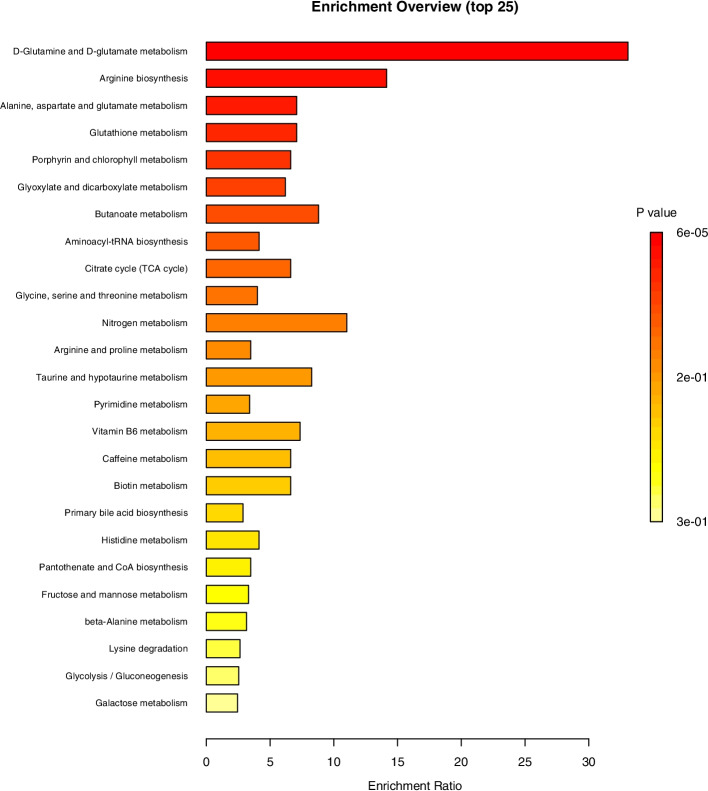
MetaboAnalyst 5.0 Pathway Enrichment Analysis for Metabolites Significantly Different Between Groups (*p* < 0.05). The enrichment ratio is calculated as the number of hits within a particular metabolic pathway divided by the expected number of hits. See the MetaboAnalyst 5.0 website for additional details: https://www.metaboanalyst.ca/docs/Faqs.xhtml#def

Figure 2 shows the MetaboAnalyst 5.0 pathway enrichment analysis used to evaluate the metabolic pathways most implicated in the results. Visualizations of our findings in relation to the citric acid cycle, which is central to energy metabolism, and the glyoxylate and dicarboxylate metabolic pathway (the metabolic pathway with the most metabolites represented in our sample) are available in Supplementary Figs. 1 and 2, respectively.

## Discussion

This study is the first to detail the metabolites, identified using untargeted metabolomics methodology, that are significantly different in adolescents with JIA in comparison with healthy controls. The metabolites were unrelated to JIA disease activity or time since diagnosis in our sample. Serine and NAA were promising differentiators between plasma samples of the JIA and control groups. Related pathways may also be promising areas of study for further research about disease prevention, pathogenesis, and management. The groups’ proportions of sex, race, and ethnicity are consistent with the general population of JIA patients in the central Texas area, and the diverse sample contained a higher percentage of racial and ethnic minorities than in samples reported from other parts of the country [31].

### Metabolites

Our findings underscore the need for sensitive, specific biomarkers that detect preclinical changes related to JIA. The plasma metabolites that differed between the JIA and control groups suggest disturbances in antioxidant metabolism and oxidative stress; neurological health; and energy, amino acid, and caffeine metabolism. We found similarities between the JIA and rheumatoid arthritis metabolomes in previous literature, but our findings also suggest differences that warrant independent investigation, prevention strategies, and treatment options for adolescents [12, 32–34].

Amino acid metabolism overall was disrupted in our JIA group. In both our JIA findings and previous studies of arthritis, most observed a decrease in circulating amino acids overall, which may reflect an increased uptake into inflamed tissue like the synovium [34–39]. Specifically, plasma serine was the strongest amino acid predictor in our samples, at 27 times lower in the JIA group than in the control group. Serine is important to brain health, intracellular energy metabolism, and antioxidant activity [40–42]. Supplementary Table 2 summarizes the existing evidence about serine metabolism in rheumatoid arthritis, osteoarthritis, and related conditions [43–49] because no prior studies were found about serine metabolism in JIA. The glycine-serine-threonine metabolic pathways were significantly enriched in previous studies that measured metabolomics in the serum, plasma, urine, and synovial fluid of animal and human models of rheumatoid arthritis [43, 44, 47, 49], osteoarthritis [46, 48], ankylosing spondylitis [49], and other related immune-mediated inflammatory diseases [43].

We consider these glycine-serine-threonine findings to be relevant for two reasons. First, metabolomics research is a frontier that is only beginning to be explored in JIA, and even though the sample is small, our results align with previous work from adults with arthritis in that amino acid metabolism is dysregulated; and that in most studies, circulating amino acid levels, such as serine, were substantially lower in JIA than in healthy controls. Because the serine was remarkably depleted across all JIA participants, despite heterogenous subtypes and medication regimens, it may be useful as a potential diagnostic biomarker. Findings should be confirmed with additional samples that are powered to detect differences between subgroups of JIA participants.

Second, these results suggest a focus on the glycine-serine-threonine metabolic pathway amongst the myriad other potential amino acids and pathways that could be studied. Since the glycine-serine-threonine pathway is involved in collagen repair in inflamed tissues, serine may be an important component of tissue repair in the synovial fluid of the inflamed joint or involved in the body’s response to systemic inflammation [35]. As such, it may be useful to study mechanisms of replenishing serine supply to ensure efficient and effective tissue repair. Additional research is warranted, perhaps incorporating multi-omics methods, to fully understand the potential therapeutic benefits of targeting the glycine-serine-threonine metabolic pathway in adolescents with JIA.

NAA, a nervous system-specific metabolite, was another strong differentiator between the JIA and control groups in our study. NAA is the second most abundant metabolite in the brain after glutamate, is a marker for neurological health, and is involved in energy metabolism in the neuronal cells [35]. Although plasma metabolites are easier and less costly to measure, most studies of NAA measure brain tissue metabolite levels using proton magnetic resonance spectroscopy, or ^1^H-MRS. [50–60] Neither brain ^1^H-MRS-measured levels of NAA nor circulating plasma concentrations of NAA have been previously described in patients with JIA. The role and function of NAA as a marker of health in the peripheral nervous system is unclear and is understudied.

Supplementary Table 3 summarizes the evidence about brain NAA levels in arthritis and related conditions like fibromyalgia or systemic lupus erythematosus. Brain NAA levels, sometimes reported as a ratio with creatinine or choline levels, were lower in those with arthritis and related disease states in comparison with healthy controls [50–60]. Low NAA, low NAA/creatinine, or low NAA/choline have previously been associated with higher disease severity and worse outcomes (i.e. death, poor cerebellar function, depression, pain, and migraine) [53–60]. Post-hoc analysis revealed that the NAA/creatinine ratio was significantly lower in adolescents with JIA than in controls, *t*(37) = 7.192, *p* < 0.0001. Choline was not detected in our samples. Future studies should investigate the relationships between NAA/creatinine and NAA/choline ratios and JIA outcomes.

NAA levels have been observed to normalize after surgical resolution of osteoarthritis, fibromyalgia flares, or post-rheumatic fever [59, 61]. The normalization response after flares of inflammation suggests a reversible mechanism linked to mitochondrial dysfunction [58]. This mechanistic theory is extended by recent work on aspartate by Wu et al. [61], although they did not measure or report on NAA directly. Wu et al. identified a reversible mitochondrial deficiency in aspartate production in the synovial fluid T-cells of arthritic joints and considered this dysfunction to be significant in arthritis pathogenesis. Given that aspartate is an amino acid, this concept aligns with our findings that amino acid metabolism overall is dysregulated in JIA. It also aligns with the suggestion that decreased levels of circulating amino acids reflect an increased uptake into inflamed synovial tissue [33, 37], perhaps in part to compensate for mitochondrial deficiency in the synovial immune cells. Since we did not detect aspartate in our samples, future targeted work that measures both aspartate and NAA, comparing synovial fluid and blood, may provide more insight. Further, multi-omics approaches that define the interactions between the production and regulation of amino acids, enzymes catalyzing the aspartate and NAA metabolic pathways, and other relevant mitochondrial proteins may provide a more complete picture of the mechanism of dysfunction.

Chronically depressed brain NAA levels may signal irreversible nerve damage and grave prognoses, as has been described in patients with fatal systemic lupus erythematosus [52]. The evidence is mixed about how circulating NAA levels may be related to brain NAA levels, and more studies need to be done [62, 63]. However, it is possible that both circulating and brain tissue NAA levels that do not normalize after an acute injury or flare may be a useful biomarker of prognosis in JIA.

The 23 metabolites found to be significantly different between groups, including those detected in only one group, collectively narrow the focus for future precision health research. Together, pathway enrichment analysis indicates that the pathways implicated by our findings are consistent with previous studies in rheumatoid arthritis [35, 38, 64, 65]. Our findings also add new information unique to adolescents with JIA.

It is important to note that the sample of adolescents in this study was representative of the self-identified Black race and Hispanic/Latinx ethnicity populations in Texas [31], groups that are historically underrepresented in clinical research [66]. As such, this study contributes beginning evidence toward future precision health research that differentiates between racial and ethnic groups. Racially and ethnically diverse samples of adolescents are essential to the generalizability of precision health research [66]. Defining the metabolome based on self-identified race or ethnicity may provide scant evidence of genomic differences since it is not a measure of genetic ancestry [67]. However, categorization based on race or ethnicity may advance our understanding of an individual’s physiologic response to their environment. This is because metabolomic characteristics are downstream from epigenomic and transcriptomic systems of differential gene expression [68]. Self-identified race and ethnicity classification may also be useful for psychosocial phenotyping as a complementary approach to precision medicine [69].

### Limitations

This snapshot of fasting morning metabolism adds new information that may potentially inform future studies. However, this was a cross-sectional study of participants at a single time point; a design with repeated measures may provide more information about how metabolites and outcomes may change over time [69]. Due to our small overall and subgroup sample sizes, the findings should be confirmed in a larger sample of adolescents.

Maturational hormones are known to affect metabolomics findings, but the literature is sparse on the specific metabolites that vary during maturation in adolescents beyond those directly related to sex hormone metabolism pathways [70, 71]. We kept the age range narrow for this study to reduce variation due to maturational stage, because it was impracticable to measure Tanner stages in our control group. To account for differences, we controlled for age and sex in the generalized linear model. All females reported onset of menarche prior to the study visit. Nonetheless, it is unknown to what degree maturation hormones contributed to the findings.

Participants in both groups reported taking multiple medications, vitamins, or supplements during the 7 days prior to their study visit. Future studies might consider standardization of medications as inclusion criteria, a washout period (when appropriate) within days prior to the study visit, or both. Future studies might consider incorporating additional comparison groups: either participants with new onset JIA who are naïve to treatment or those with JIA in remission who are not taking any medications could be compared with heathy controls, or one could use a three-group design.

Finally, there are limitations inherent to mass spectrometry analysis of plasma samples. Circulating metabolites in the plasma are not direct measures of mitochondrial or intracellular metabolism. To fully understand any mitochondrial dysfunction, additional studies should be conducted with targeted metabolomics. Untargeted analysis does not allow us to provide absolute concentrations of metabolites in the blood. Additional validation studies will be required before new metabolite biomarkers are appropriate for clinical use.

## Conclusions

In this study, we have identified a distinct set of metabolites in adolescents with JIA relative to controls after adjusting for age, sex, and medications or supplements. The study sample was more representative of the general population than samples in prior research, with more non-White diversity than in previous metabolomic studies of adolescents with JIA. Serine and NAA were sensitive and specific differentiators of JIA and control group membership, and they hold promise as candidate biomarkers for further research. The implicated pathways suggest differences in oxidative stress, neurological health, energy, caffeine, and amino acid metabolism.

Our findings support continued study with a larger sample, because our results with adolescents were both similar to yet different from those reported previously in adults with rheumatoid arthritis. Future studies should expand the age groups to include all children diagnosed with JIA to fully describe the metabolites across maturation stages and should incorporate more new-onset JIA patients who are in active disease and naïve to treatment. In addition, future research should consider a three-group repeated measures design that incorporates patients naïve to treatment compared with controls and adolescents with inactive disease. Finally, the integration of the plasma metabolome findings with genomics, transcriptomics, or epigenomics may further elucidate sources of pathway disruption to advance our understanding of systems biology. The findings of this study indicate that sensitive, specific measures of JIA for racially and ethnically diverse adolescents are still needed, and that metabolomics may be a promising methodology for this purpose.

## Supplementary Information

**Additional file 1: Supplementary File 1.** R Code Used for Statistical Analyses.

**Additional file 2: Supplementary Fig. 1.** Citrate Cycle Comparing Plasma Metabolites by Group: Juvenile Idiopathic Arthritis Relative to Controls.

**Additional file 3: Supplementary Fig. 2.** Glyoxylate and Dicarboxylate Pathway Comparing Plasma Metabolites: Juvenile Idiopathic Arthritis Relative to Controls.

**Additional file 4: Supplementary Table 1.** Reported Intake of One or More Medications or Supplements within Each Category within 7 Days Prior to Study Visit. **Supplementary Table 2.** Overview of Literature about the Role of Serine Metabolism in Arthritis and Related Conditions (plasma serine or serum serine or circulating serine or L-serine) AND (arthritis) AND (metabolome or metabolomics or metabolite). **Supplementary Table 3.** Overview of Brain Tissue *N*-Acetylaspartate Literature about Arthritis and Related Diseases.

## Data Availability

The dataset supporting the conclusions of this article is available in the Zenodo repository, 10.5281/zenodo.5834651.

## Abbreviations

BMI: Body mass index
^1^H-MRS: Proton magnetic resonance spectroscopy
JIA: Juvenile idiopathic arthritis
NAA: *N*-acetylaspartate or *N*-acetyl-aspartic acid
