# Supplementary material for: Serine, N-acetylaspartate differentiate adolescents with juvenile idiopathic arthritis compared with healthy controls: a metabolomics cross-sectional study
Source: Pediatr Rheumatol Online J. 2022 Feb 10;20:12. doi: 10.1186/s12969-022-00672-z (PMC8832851; doi:10.1186/s12969-022-00672-z)

Citrate Cycle (TCA Cycle)

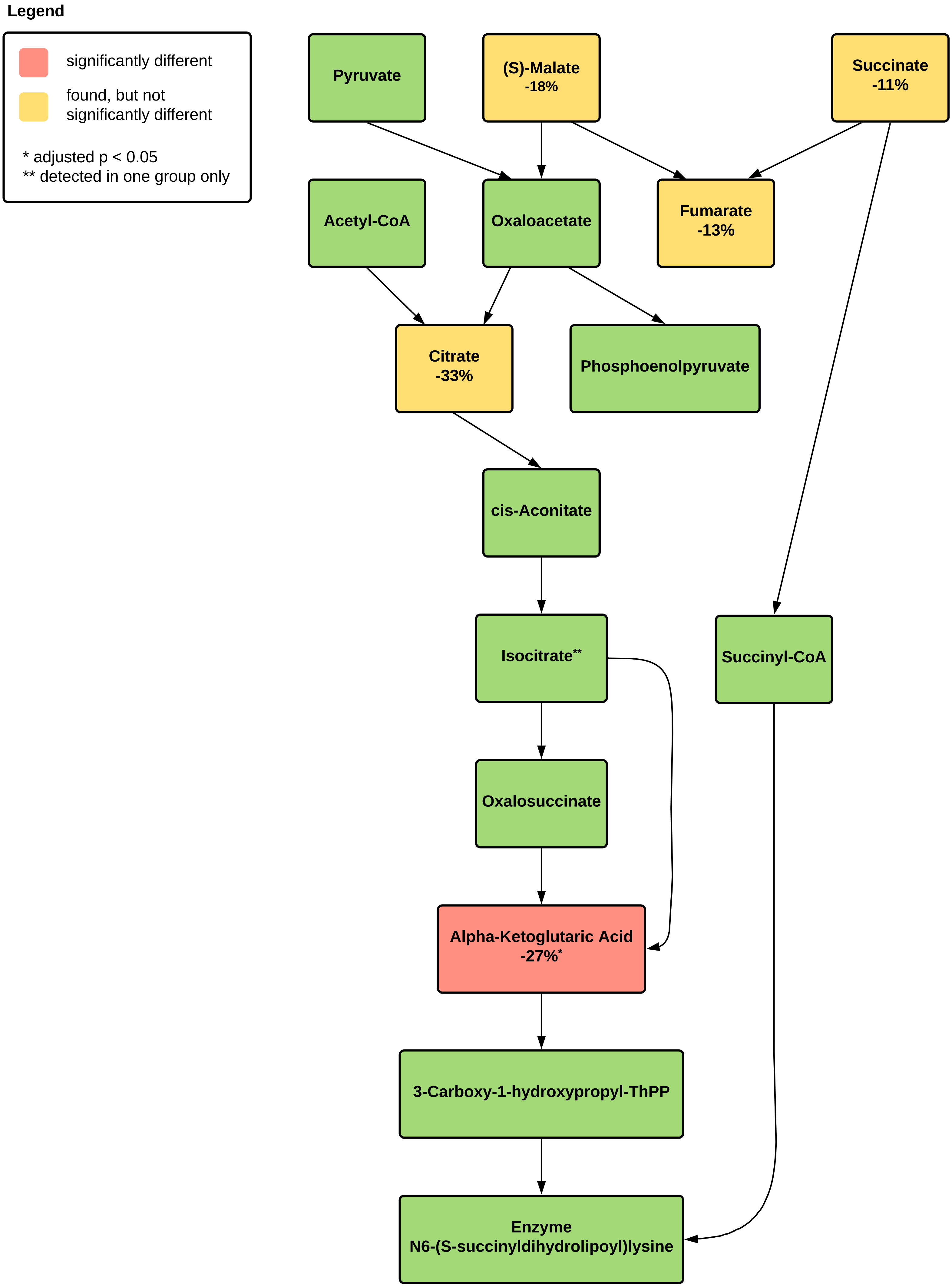

Supplement: Supplementary file 1 — Additional file 1: Supplementary File 1. R Code Used for Statistical Analyses. [file 12969_2022_672_MOESM1_ESM.pdf]
