# Supplementary material for: Serine, N-acetylaspartate differentiate adolescents with juvenile idiopathic arthritis compared with healthy controls: a metabolomics cross-sectional study
Source: Pediatr Rheumatol Online J. 2022 Feb 10;20:12. doi: 10.1186/s12969-022-00672-z (PMC8832851; doi:10.1186/s12969-022-00672-z)

Glyoxylate and Dicarboxylate Metabolism

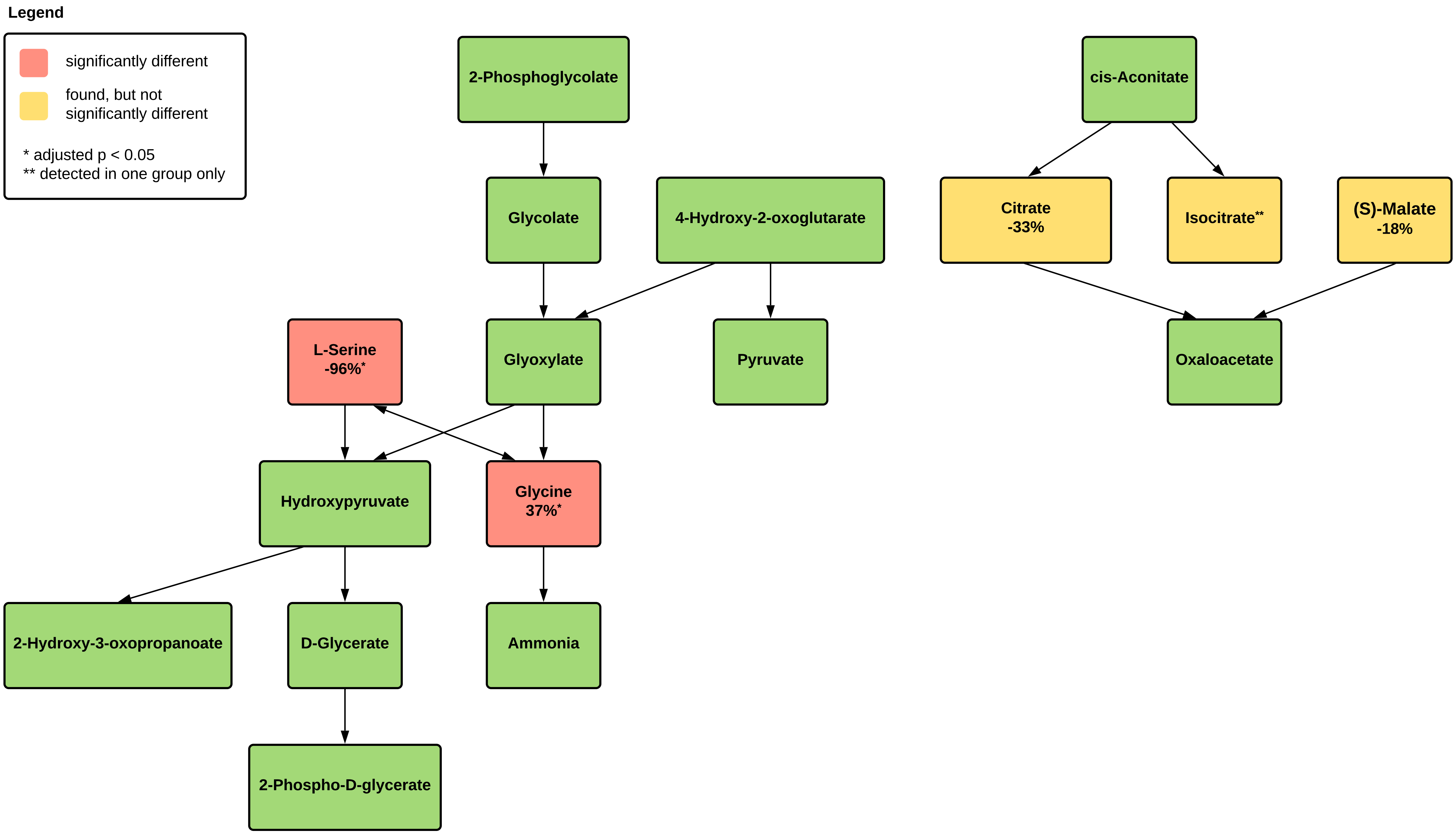

Supplement: Supplementary file 2 — Additional file 2: Supplementary Fig. 1. Citrate Cycle Comparing Plasma Metabolites by Group: Juvenile Idiopathic Arthritis Relative to Controls. [file 12969_2022_672_MOESM2_ESM.pdf]
