# Supplementary material for: Serine, N-acetylaspartate differentiate adolescents with juvenile idiopathic arthritis compared with healthy controls: a metabolomics cross-sectional study
Source: Pediatr Rheumatol Online J. 2022 Feb 10;20:12. doi: 10.1186/s12969-022-00672-z (PMC8832851; doi:10.1186/s12969-022-00672-z)
Supplement: Supplementary file 3 — Additional file 3: Supplementary Fig. 2. Glyoxylate and Dicarboxylate Pathway Comparing Plasma Metabolites: Juvenile Idiopathic Arthritis Relative to Controls. [file 12969_2022_672_MOESM3_ESM.pdf]

JIA Analysis

EDG

5/5/2021

Contents

Setup **1**
Libraries . . . 1
Data . . . 1
Scale . . . 1

Metabolite Group Differences: T-tests **1**

Metabolite Group Differences with covariates: GLM **3**
Plot . . . 5

Metabolite Levels vs. Time Since Diagnosis **6**
Pearson's r . . . 7
Spearman's rho . . . 8

Metabolite Levels vs. Physician Global Assessment **10**
Pearson's r . . . 10
Spearman's rho . . . 11

Setup

Libraries

Data

```
datsn <- readRDS("../Data/datsn.rds")
```

Scale

```
met_scaled <- as.data.frame(scale(datsn[, 26:80]))
```

Metabolite Group Differences: T-tests

T-tests with Holm correction for multiple comparisons

```r
jia_met_ttest <- lapply(seq(met_scaled), \(i) t.test(met_scaled[, i] ~ datsn$Group))
jia_met_ttest_summary <- data.frame(Metabolite = names(met_scaled))
jia_met_ttest_summary <- cbind(jia_met_ttest_summary,
                               t(sapply(jia_met_ttest, \(i)
                                        data.frame(`t-statistic` = i$statistic,
                                                   `p-value` = i$p.value))))
jia_met_ttest_summary$`Holm-adjusted p-value` <- p.adjust(jia_met_ttest_summary$p.value,
                                                          method = "holm")
jia_met_ttest_summary$`FDR-adjusted p-value` <- p.adjust(jia_met_ttest_summary$p.value,
                                                         method = "fdr")
kable(jia_met_ttest_summary, align = "r", row.names = FALSE)
```

Metabolite	t.statistic	p.value	Holm-adjusted p-value	FDR-adjusted p-value
Lactate	2.186901	0.0357373	1.0000000	0.1156207
Malate	3.529327	0.001177516	0.0565207	0.0080954
3-Hydroxykynurenine	0.7184789	0.4778709	1.0000000	0.7288919
4-Guanidinobutanoate	-0.02011001	0.9840745	1.0000000	0.9840745
5-6-Dihydrouracil	0.2016787	0.8413334	1.0000000	0.9129293
5-Methyl-Cytosine	0.6297059	0.5327573	1.0000000	0.7288919
5-Oxolproline	0.6932582	0.4928784	1.0000000	0.7288919
Adenine	-0.257635	0.7983243	1.0000000	0.9052649
Alpha-D-Glucose	4.127766	0.0002843546	0.0142177	0.0026066
Alpha-Ketoglutaric_Acid	5.020581	1.325711e-05	0.0007026	0.0002430
Beta-Alanine	0.4311453	0.6688779	1.0000000	0.8555415
Betaine	0.6744346	0.504399	1.0000000	0.7288919
Citrate	4.811946	2.71169e-05	0.0014101	0.0003729
Citrulline	2.453104	0.01904446	0.8189117	0.0805727
Creatine	-0.2671628	0.7908291	1.0000000	0.9052649
Creatinine	0.1459697	0.8847558	1.0000000	0.9153440
Cytidine	-1.071919	0.2907375	1.0000000	0.5330187
Deoxycarnitine	-0.7896664	0.4347554	1.0000000	0.7245923
D-Pantothenic_Acid	-0.2997811	0.7663515	1.0000000	0.9052649
D-Ribose-5-Phosphate	0.1949261	0.8465345	1.0000000	0.9129293
Fumarate	1.428042	0.1616806	1.0000000	0.3488861
Glycerol	1.458751	0.1531101	1.0000000	0.3488861
Glycine	-3.71795	0.001000909	0.0490446	0.0078643
Guanosine	-0.1281821	0.8987014	1.0000000	0.9153440
Homocysteine	1.115475	0.2719664	1.0000000	0.5330187
Inosine	-0.1339504	0.8941768	1.0000000	0.9153440
L-Arginine	-0.3494344	0.7289721	1.0000000	0.8909659
L-Asparagine	-0.5303817	0.5990164	1.0000000	0.7844262
L-Carnitine	-1.085005	0.2859545	1.0000000	0.5330187
L-Cystine	-4.435975	0.0001642153	0.0083750	0.0018064
L-Eucine	1.576255	0.1267853	1.0000000	0.3169632
L-Glutamine	1.416845	0.164928	1.0000000	0.3488861
L-Histidine	2.237901	0.03171576	1.0000000	0.1090229
L-Isoleucine	0.6989755	0.4895074	1.0000000	0.7288919
L-Kynurenine	0.6440178	0.5263491	1.0000000	0.7288919
L-Methionine	1.672433	0.1030556	1.0000000	0.2950846
L-Phenylalanine	2.724009	0.009857846	0.4337452	0.0451818
L-Proline	2.356714	0.0245927	1.0000000	0.0966142
L-Serine	26.47496	9.336695e-17	0.0000000	0.0000000
L-Threonine	0.6142679	0.5433558	1.0000000	0.7288919

Metabolite	t.statistic	p.value	Holm-adjusted p-value	FDR-adjusted p-value
L-Tyrosine	2.296675	0.02740581	1.0000000	0.1004880
Myristic_acid	3.223378	0.002688569	0.1236742	0.0147871
Methyl-L-Histidine	-1.281818	0.2082531	1.0000000	0.4242194
N-Acetyl-DL-Methionine	-0.3649125	0.7180391	1.0000000	0.8909659
N-Acetyl-DL-Serine	2.83829	0.007385132	0.3323309	0.0369257
N-Acetylglycine	0.6781774	0.5023467	1.0000000	0.7288919
N-Acetyl-L-Aspartic_Acid	13.19133	9.257062e-14	0.0000000	0.0000000
N-Acetylputrescine	1.491702	0.1447816	1.0000000	0.3462168
Acetyllcarnitine	1.619881	0.1139611	1.0000000	0.2984696
Pipecolate	0.8480414	0.4035521	1.0000000	0.6936051
Spermidine	-0.2466971	0.8065087	1.0000000	0.9052649
Succinate	1.650792	0.1073035	1.0000000	0.2950846
Transaconitate	2.048311	0.04793854	1.0000000	0.1464789
Uracil	3.369733	0.001778222	0.0835764	0.0108669
Urocanate	0.8700457	0.390216	1.0000000	0.6923187

Which metabolites have significant t-test p-values after correction?

Holm-adjusted:

```
sigholm_ttest <- which(jia_met_ttest_summary$`Holm-adjusted p-value` < .05)
jia_met_ttest_summary$Metabolite[sigholm_ttest]
```

```
[1] "Alpha-D-Glucose"       "Alpha-Ketoglutaric_Acid"  "Citrate"                 "Glycine"
[5] "L-Cystine"               "L-Serine"                "N-Acetyl-L-Aspartic_Acid"
```

FDR-adjusted:

```
sigfdr_ttest <- which(jia_met_ttest_summary$`FDR-adjusted p-value` < .05)
jia_met_ttest_summary$Metabolite[sigfdr_ttest]
```

```
 [1] "Malate"                "Alpha-D-Glucose"       "Alpha-Ketoglutaric_Acid"  "Citrate"
 [5] "Glycine"               "L-Cystine"            "L-Phenylalanine"        "L-Serine"
 [9] "Myristic_acid"           "N-Acetyl-DL-Serine"      "N-Acetyl-L-Aspartic_Acid" "Uracil"
```

Metabolite Group Differences with covariates: GLM

```
jia_met_glm <- lapply(seq(met_scaled), \(i) glm(met_scaled[, i] ~ datsn$Group +
                                                  datsn$Age + datsn$Sex +
                                                  datsn$Meds_Rheum + datsn$Meds_Nonrheum,
                                                family = "gaussian"))
names(jia_met_glm) <- names(met_scaled)
jia_met_glm_summary <- data.frame(Metabolite = names(met_scaled))
jia_met_glm_summary <- cbind(jia_met_glm_summary,
                             t(sapply(jia_met_glm, \(i) {
                               .GroupJIA <- summary(i)$coefficient[2, ]
                                      data.frame(`Coefficient` = .GroupJIA[["Estimate"]],
                                                 `p-value` = .GroupJIA[["Pr(>|t|)"]])
                             })))
jia_met_glm_summary$`Holm-adjusted p-value` <- p.adjust(jia_met_glm_summary$p.value, method =
```

```
jia_met_glm_summary$`FDR-adjusted p-value` <- p.adjust(jia_met_glm_summary$p.value, method = "
kable(jia_met_glm_summary, align = "r", row.names = FALSE, digits = 4)
```

Metabolite	Coefficient	p.value	Holm-adjusted p-value	FDR-adjusted p-value
Lactate	-0.6903195	0.1689952	1.0000	0.3718
Malate	-1.200449	0.005422104	0.2603	0.0373
3-Hydroxykynurenine	-1.084635	0.02780372	1.0000	0.1019
4-Guanidinobutanoate	-0.5691045	0.2266426	1.0000	0.4617
5-6-Dihydrouracil	0.2882152	0.588212	1.0000	0.7637
5-Methyl-Cytosine	-0.7446801	0.1611632	1.0000	0.3693
5-Oxolproline	-0.103517	0.8446467	1.0000	0.8934
Adenine	-0.2710157	0.5970971	1.0000	0.7637
Alpha-D-Glucose	-1.628646	0.0002877638	0.0150	0.0040
Alpha-Ketoglutaric_Acid	-1.813999	3.447593e-05	0.0018	0.0006
Beta-Alanine	-0.2090596	0.6933903	1.0000	0.8303
Betaine	-0.3611235	0.4957941	1.0000	0.7049
Citrate	-1.334746	0.001775516	0.0870	0.0140
Citrulline	-0.7730851	0.09645386	1.0000	0.2526
Creatine	0.8515288	0.08270359	1.0000	0.2526
Creatinine	-0.1972678	0.6200222	1.0000	0.7750
Cytidine	0.3791109	0.3772907	1.0000	0.6288
Deoxycarnitine	-0.03266616	0.9460577	1.0000	0.9636
D-Pantothenic_Acid	-0.2790021	0.5893091	1.0000	0.7637
D-Ribose-5-Phosphate	-0.4031286	0.4345868	1.0000	0.6640
Fumarate	-0.8062942	0.09251522	1.0000	0.2526
Glycerol	-0.4127435	0.3650058	1.0000	0.6274
Glycine	1.56173	0.000411657	0.0210	0.0045
Guanosine	0.1381015	0.7767331	1.0000	0.8718
Homocysteine	-0.1908088	0.7014652	1.0000	0.8303
Inosine	0.01717053	0.9724852	1.0000	0.9725
L-Arginine	-0.1499764	0.7580897	1.0000	0.8686
L-Asparagine	0.8033012	0.09613341	1.0000	0.2526
L-Carnitine	0.8559752	0.08949029	1.0000	0.2526
L-Cystine	1.602693	0.0004920382	0.0246	0.0045
L-Eucine	-1.227584	0.01378623	0.6204	0.0672
L-Glutamine	-0.4288286	0.4068472	1.0000	0.6581
L-Histidine	-0.3652667	0.4470324	1.0000	0.6645
L-Isoleucine	-0.5439145	0.2991594	1.0000	0.5485
L-Kynurenine	-1.125342	0.02531739	1.0000	0.0995
L-Methionine	-0.5562826	0.2778748	1.0000	0.5458
L-Phenylalanine	-1.224782	0.01049856	0.4934	0.0642
L-Proline	-0.3844127	0.4326505	1.0000	0.6640
L-Serine	-1.90878	5.90479e-17	0.0000	0.0000
L-Threonine	0.3280403	0.4998067	1.0000	0.7049
L-Tyrosine	-0.4780833	0.3485121	1.0000	0.6183
Myristic_acid	-0.463609	0.2953753	1.0000	0.5485
Methyl-L-Histidine	0.1263642	0.8058513	1.0000	0.8864
N-Acetyl-DL-Methionine	-0.06068125	0.9075169	1.0000	0.9418
N-Acetyl-DL-Serine	-1.203735	0.01224508	0.5633	0.0672
N-Acetylglycine	-0.7288815	0.1571267	1.0000	0.3693
N-Acetyl-L-Aspartic_Acid	-1.981372	1.375209e-10	0.0000	0.0000
N-Acetylputrescine	-0.8780158	0.0801201	1.0000	0.2526
Acetyllcarnitine	-0.7244277	0.1191086	1.0000	0.2978

Metabolite	Coefficient	p.value	Holm-adjusted p-value	FDR-adjusted p-value
Pipecolate	-0.174792	0.7095356	1.0000	0.8303
Spermidine	-0.3332325	0.5130738	1.0000	0.7055
Succinate	-0.6194766	0.1867588	1.0000	0.3951
Transaconitate	-1.125657	0.01589317	0.6834	0.0672
Uracil	-1.18752	0.01527213	0.6720	0.0672
Urocanate	0.1059911	0.84103	1.0000	0.8934

Which metabolites have significant coefficient p-values after correction?

Holm-adjusted:

```
sigholm_glm <- which(jia_met_glm_summary$`Hold-adjusted p-value` < .05)
jia_met_glm_summary$Metabolite[sig_glm]
```

```
[1] "Alpha-D-Glucose"       "Alpha-Ketoglutaric_Acid"  "Glycine"              "L-Cystine"
[5] "L-Serine"                  "N-Acetyl-L-Aspartic_Acid"
```

FDR-adjusted:

```
sigfdr_glm <- which(jia_met_glm_summary$`FDR-adjusted p-value` < .05)
jia_met_glm_summary$Metabolite[sigfdr_glm]
```

```
[1] "Malate"                "Alpha-D-Glucose"       "Alpha-Ketoglutaric_Acid"  "Citrate"
[5] "Glycine"               "L-Cystine"             "L-Serine"                 "N-Acetyl-L-Aspartic_Acid"
```

Plot

Box plots of metabolites with significant group coefficients

```
rtlayout(6, 1)
for (i in sig_glm) {
  mplot3.box(split(met_scaled[, i], datsn$Group),
             main = labelify(names(met_scaled[i])),
             xlab = NA,
             mar = c(1.4, 4, 1.6, .5),
             horizontal = TRUE)
}
```

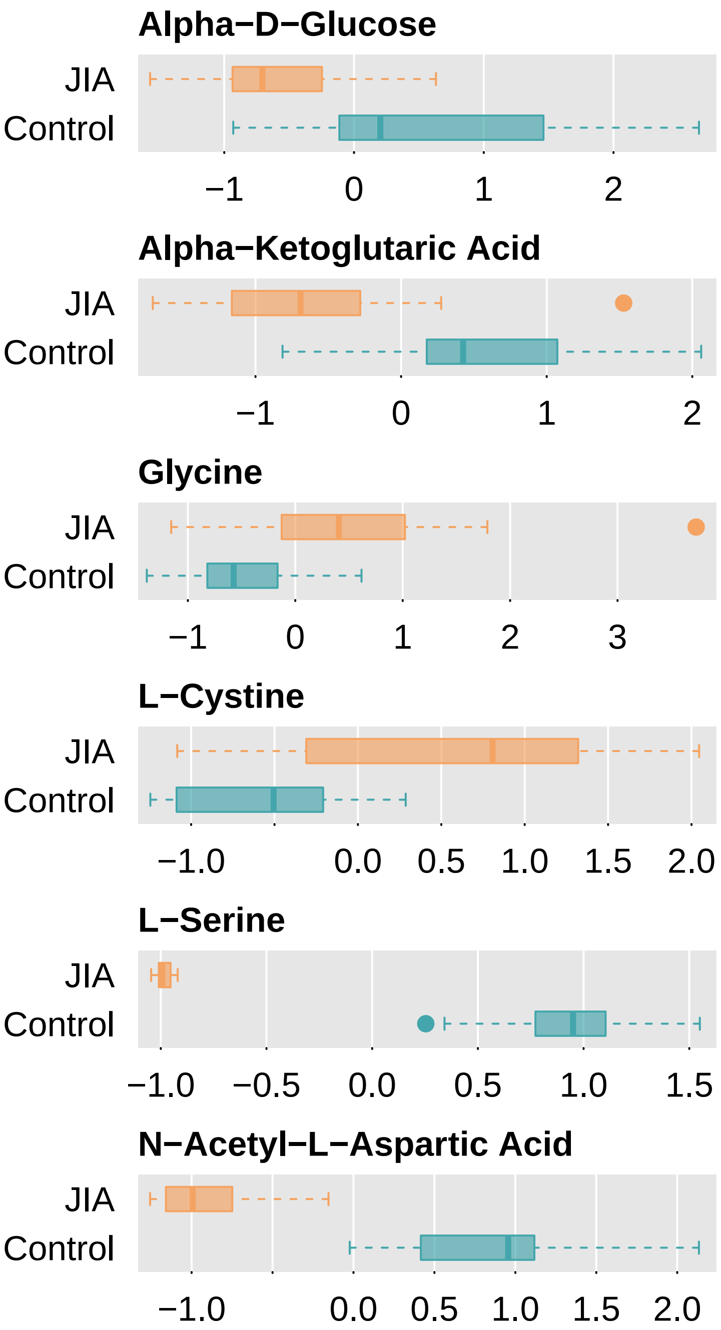


```
rtlayout()
```

Metabolite Levels vs. Time Since Diagnosis

```
index_jia <- datsn$Group == "JIA"
```

Pearson's r

```
jia_met_time_pearson <- lapply(seq(met_scaled),
                                \(i) cor.test(met_scaled[index_jia, i],
                                              datsn$Time_Since_Diagnosis[index_jia],
                                              method = "pearson"))
jia_met_time_pearson_summary <- data.frame(Metabolite = names(met_scaled))
jia_met_time_pearson_summary <- cbind(jia_met_time_pearson_summary,
                               t(sapply(jia_met_time_pearson, \(i)
                                        data.frame(`Pearson's r` = i$statistic,
                                                   `p-value` = i$p.value))))
jia_met_time_pearson_summary$`Adjusted p-value` <-
  p.adjust(jia_met_time_pearson_summary$p.value, method = "holm")
kable(jia_met_time_pearson_summary, align = "r", row.names = FALSE)
```

Metabolite	Pearson.s.r	p.value	Adjusted p-value
Lactate	0.4212928	0.6788198	1.0000000
Malate	1.386369	0.1835493	1.0000000
3-Hydroxykynurenine	0.230608	0.8203717	1.0000000
4-Guanidinobutanoate	-0.004258906	0.9966515	1.0000000
5-6-Dihydrouracil	-0.6905045	0.499197	1.0000000
5-Methyl-Cytosine	0.5305116	0.6026192	1.0000000
5-Oxolproline	-0.5871436	0.5648245	1.0000000
Adenine	-1.852346	0.08142102	1.0000000
Alpha-D-Glucose	-0.3498878	0.7307201	1.0000000
Alpha-Ketoglutaric_Acid	0.8881595	0.3868492	1.0000000
Beta-Alanine	-0.745945	0.4658911	1.0000000
Betaine	2.228967	0.03959736	1.0000000
Citrate	0.7555076	0.4602851	1.0000000
Citrulline	1.085472	0.2928661	1.0000000
Creatine	-0.04720437	0.9629005	1.0000000
Creatinine	-0.697333	0.4950215	1.0000000
Cytidine	-0.9709861	0.3451688	1.0000000
Deoxycarnitine	2.291718	0.03495995	1.0000000
D-Pantothenic_Acid	-0.515509	0.6128363	1.0000000
D-Ribose-5-Phosphate	0.7285078	0.4762194	1.0000000
Fumarate	-0.2065519	0.8388135	1.0000000
Glycerol	1.778088	0.09327977	1.0000000
Glycine	-1.170332	0.2580129	1.0000000
Guanosine	-0.3944407	0.6981601	1.0000000
Homocysteine	-1.208516	0.2433911	1.0000000
Inosine	-0.4535935	0.6558582	1.0000000
L-Arginine	1.039705	0.3130391	1.0000000
L-Asparagine	1.055179	0.3061097	1.0000000
L-Carnitine	0.05194707	0.9591764	1.0000000
L-Cystine	-1.019774	0.3221297	1.0000000
L-Eucine	0.7974545	0.4361847	1.0000000
L-Glutamine	0.1255097	0.9015924	1.0000000
L-Histidine	-2.224383	0.03995754	1.0000000
L-Isoleucine	0.4068114	0.6892225	1.0000000
L-Kynurenine	0.81042	0.4288985	1.0000000
L-Methionine	0.6609769	0.5174854	1.0000000
L-Phenylalanine	1.262137	0.223941	1.0000000

Metabolite	Pearson.s.r	p.value	Adjusted p-value
L-Proline	-0.6743488	0.5091567	1.0000000
L-Serine	0.2315568	0.8196465	1.0000000
L-Threonine	-0.8597396	0.4018944	1.0000000
L-Tyrosine	-0.6366414	0.5328381	1.0000000
Myristic_acid	-3.792096	0.001455611	0.0800586
Methyl-L-Histidine	0.09310432	0.9269091	1.0000000
N-Acetyl-DL-Methionine	-0.5649823	0.5794659	1.0000000
N-Acetyl-DL-Serine	-0.07321936	0.9424862	1.0000000
N-Acetylglycine	1.077057	0.2965022	1.0000000
N-Acetyl-L-Aspartic_Acid	-0.2947842	0.7717282	1.0000000
N-Acetylputrescine	-1.606755	0.1265217	1.0000000
Acetyllcarnitine	2.088299	0.05212599	1.0000000
Pipecolate	-1.455369	0.1637853	1.0000000
Spermidine	-0.1228579	0.9036604	1.0000000
Succinate	0.7075967	0.4887839	1.0000000
Transaconitate	0.0178729	0.9859483	1.0000000
Uracil	-0.1177506	0.9076453	1.0000000
Urocanate	-1.097814	0.2875924	1.0000000

```
which(jia_met_time_pearson_summary$`Adjust p-value` < .05)
```

```
integer(0)
```

Spearman's rho

```
jia_met_time_spearman <- lapply(seq(met_scaled),
                                \(i) suppressWarnings(
                                  cor.test(met_scaled[index_jia, i],
                                           datsn$Time_Since_Diagnosis[index_jia],
                                           method = "spearman")))
jia_met_time_spearman_summary <- data.frame(Metabolite = names(met_scaled))
jia_met_time_spearman_summary <- cbind(jia_met_time_spearman_summary,
                               t(sapply(jia_met_time_spearman, \(i)
                                        data.frame(`spearman's r` = i$statistic,
                                                   `p-value` = i$p.value))))
jia_met_time_spearman_summary$`Adjusted p-value` <-
  p.adjust(jia_met_time_spearman_summary$p.value, method = "holm")
kable(jia_met_time_spearman_summary, align = "r", row.names = FALSE)
```

Metabolite	spearman.s.r	p.value	Adjusted p-value
Lactate	1031.667	0.698772	1
Malate	767.8557	0.1725532	1
3-Hydroxykynurenine	1072.793	0.8105369	1
4-Guanidinobutanoate	1287.453	0.5976715	1
5-6-Dihydrouracil	1256.358	0.6775638	1
5-Methyl-Cytosine	999.5682	0.6153708	1
5-Oxolproline	1257.361	0.6749287	1
Adenine	1684.675	0.03855387	1
Alpha-D-Glucose	1211.219	0.7994389	1

Metabolite	spearman.s.r	p.value	Adjusted p-value
Alpha-Ketoglutaric_Acid	858.1333	0.3074748	1
Beta-Alanine	1363.688	0.4207626	1
Betaine	602.3468	0.041496	1
Citrate	879.1981	0.3461523	1
Citrulline	913.3029	0.4144138	1
Creatine	979.5065	0.5653745	1
Creatinine	1503.117	0.1838198	1
Cytidine	1220.247	0.7746057	1
Deoxycarnitine	598.3344	0.03979441	1
D-Pantothenic_Acid	1069.784	0.8022101	1
D-Ribose-5-Phosphate	947.4078	0.4893162	1
Fumarate	1122.948	0.9515359	1
Glycerol	587.3005	0.03539137	1
Glycine	1477.036	0.2191074	1
Guanosine	1300.494	0.5653745	1
Homocysteine	1223.256	0.766373	1
Inosine	1243.318	0.7121377	1
L-Arginine	979.5065	0.5653745	1
L-Asparagine	996.5589	0.6077597	1
L-Carnitine	1030.664	0.6961089	1
L-Cystine	1496.095	0.1929174	1
L-Eucine	931.3584	0.453272	1
L-Glutamine	1229.275	0.7499802	1
L-Histidine	1685.678	0.03814706	1
L-Isoleucine	1050.725	0.7499802	1
L-Kynurenine	884.2135	0.3557579	1
L-Methionine	1071.79	0.8077591	1
L-Phenylalanine	709.6768	0.1110978	1
L-Proline	1255.355	0.6802026	1
L-Serine	1090.849	0.8608784	1
L-Threonine	1480.046	0.2148256	1
L-Tyrosine	1270.401	0.6410103	1
Myristic_acid	1745.863	0.01919564	1
Methyl-L-Histidine	1067.778	0.7966701	1
N-Acetyl-DL-Methionine	1224.259	0.7636341	1
N-Acetyl-DL-Serine	1321.558	0.5148828	1
N-Acetylglycine	846.0963	0.2865936	1
N-Acetyl-L-Aspartic_Acid	1468.009	0.2322848	1
N-Acetylputrescine	1481.049	0.2134106	1
Acetyllcarnitine	547.1771	0.02247344	1
Pipecolate	1482.052	0.2120018	1
Spermidine	1304.506	0.5555929	1
Succinate	888.2258	0.3635512	1
Transaconitate	1051.729	0.7527054	1
Uracil	1108.904	0.9117391	1
Urocanate	1222.253	0.7691146	1

```
which(jia_met_time_spearman_summary$`Adjust p-value` < .05)
```

```
integer(0)
```

No correlations between metabolite levels and time since diagnosis are significant

Metabolite Levels vs. Physician Global Assessment

Pearson's r

```
jia_met_global_pearson <- lapply(seq(met_scaled),
                                \(i) cor.test(met_scaled[index_jia, i],
                                              datsn$Physician_Global_Assessment[index_jia],
                                              method = "pearson"))
jia_met_global_pearson_summary <- data.frame(Metabolite = names(met_scaled))
jia_met_global_pearson_summary <- cbind(jia_met_global_pearson_summary,
                               t(sapply(jia_met_time_pearson, \(i)
                                        data.frame(`Pearson's r` = i$statistic,
                                                   `p-value` = i$p.value))))
jia_met_global_pearson_summary$`Adjusted p-value` <-
  p.adjust(jia_met_global_pearson_summary$p.value, method = "holm")
kable(jia_met_global_pearson_summary, align = "r", row.names = FALSE)
```

Metabolite	Pearson.s.r	p.value	Adjusted p-value
Lactate	0.4212928	0.6788198	1.0000000
Malate	1.386369	0.1835493	1.0000000
3-Hydroxykynurenine	0.230608	0.8203717	1.0000000
4-Guanidinobutanoate	-0.004258906	0.9966515	1.0000000
5-6-Dihydrouracil	-0.6905045	0.499197	1.0000000
5-Methyl-Cytosine	0.5305116	0.6026192	1.0000000
5-Oxolproline	-0.5871436	0.5648245	1.0000000
Adenine	-1.852346	0.08142102	1.0000000
Alpha-D-Glucose	-0.3498878	0.7307201	1.0000000
Alpha-Ketoglutaric_Acid	0.8881595	0.3868492	1.0000000
Beta-Alanine	-0.745945	0.4658911	1.0000000
Betaine	2.228967	0.03959736	1.0000000
Citrate	0.7555076	0.4602851	1.0000000
Citrulline	1.085472	0.2928661	1.0000000
Creatine	-0.04720437	0.9629005	1.0000000
Creatinine	-0.697333	0.4950215	1.0000000
Cytidine	-0.9709861	0.3451688	1.0000000
Deoxycarnitine	2.291718	0.03495995	1.0000000
D-Pantothenic_Acid	-0.515509	0.6128363	1.0000000
D-Ribose-5-Phosphate	0.7285078	0.4762194	1.0000000
Fumarate	-0.2065519	0.8388135	1.0000000
Glycerol	1.778088	0.09327977	1.0000000
Glycine	-1.170332	0.2580129	1.0000000
Guanosine	-0.3944407	0.6981601	1.0000000
Homocysteine	-1.208516	0.2433911	1.0000000
Inosine	-0.4535935	0.6558582	1.0000000
L-Arginine	1.039705	0.3130391	1.0000000
L-Asparagine	1.055179	0.3061097	1.0000000
L-Carnitine	0.05194707	0.9591764	1.0000000
L-Cystine	-1.019774	0.3221297	1.0000000
L-Eucine	0.7974545	0.4361847	1.0000000
L-Glutamine	0.1255097	0.9015924	1.0000000
L-Histidine	-2.224383	0.03995754	1.0000000
L-Isoleucine	0.4068114	0.6892225	1.0000000

Metabolite	Pearson.s.r	p.value	Adjusted p-value
L-Kynurenine	0.81042	0.4288985	1.0000000
L-Methionine	0.6609769	0.5174854	1.0000000
L-Phenylalanine	1.262137	0.223941	1.0000000
L-Proline	-0.6743488	0.5091567	1.0000000
L-Serine	0.2315568	0.8196465	1.0000000
L-Threonine	-0.8597396	0.4018944	1.0000000
L-Tyrosine	-0.6366414	0.5328381	1.0000000
Myristic_acid	-3.792096	0.001455611	0.0800586
Methyl-L-Histidine	0.09310432	0.9269091	1.0000000
N-Acetyl-DL-Methionine	-0.5649823	0.5794659	1.0000000
N-Acetyl-DL-Serine	-0.07321936	0.9424862	1.0000000
N-Acetylglycine	1.077057	0.2965022	1.0000000
N-Acetyl-L-Aspartic_Acid	-0.2947842	0.7717282	1.0000000
N-Acetylputrescine	-1.606755	0.1265217	1.0000000
Acetyllcarnitine	2.088299	0.05212599	1.0000000
Pipecolate	-1.455369	0.1637853	1.0000000
Spermidine	-0.1228579	0.9036604	1.0000000
Succinate	0.7075967	0.4887839	1.0000000
Transaconitate	0.0178729	0.9859483	1.0000000
Uracil	-0.1177506	0.9076453	1.0000000
Urocanate	-1.097814	0.2875924	1.0000000

```
which(jia_met_global_pearson_summary$`Adjusted p-value` < .05)
```

```
integer(0)
```

Spearman's rho

```
jia_met_global_spearman <- lapply(seq(met_scaled),
                                \(i) suppressWarnings(
                                  cor.test(met_scaled[index_jia, i],
                                           datsn$Physician_Global_Assessment[index_jia],
                                           method = "spearman")))
jia_met_global_spearman_summary <- data.frame(Metabolite = names(met_scaled))
jia_met_global_spearman_summary <- cbind(jia_met_global_spearman_summary,
                               t(sapply(jia_met_time_spearman, \(i)
                                        data.frame(`spearman's r` = i$statistic,
                                                   `p-value` = i$p.value))))
jia_met_global_spearman_summary$`Adjusted p-value` <-
  p.adjust(jia_met_global_spearman_summary$p.value, method = "holm")
kable(jia_met_global_spearman_summary, align = "r", row.names = FALSE)
```

Metabolite	spearman.s.r	p.value	Adjusted p-value
Lactate	1031.667	0.698772	1
Malate	767.8557	0.1725532	1
3-Hydroxykynurenine	1072.793	0.8105369	1
4-Guanidinobutanoate	1287.453	0.5976715	1
5-6-Dihydrouracil	1256.358	0.6775638	1
5-Methyl-Cytosine	999.5682	0.6153708	1

Metabolite	spearman.s.r	p.value	Adjusted p-value
5-Oxolproline	1257.361	0.6749287	1
Adenine	1684.675	0.03855387	1
Alpha-D-Glucose	1211.219	0.7994389	1
Alpha-Ketoglutaric_Acid	858.1333	0.3074748	1
Beta-Alanine	1363.688	0.4207626	1
Betaine	602.3468	0.041496	1
Citrate	879.1981	0.3461523	1
Citrulline	913.3029	0.4144138	1
Creatine	979.5065	0.5653745	1
Creatinine	1503.117	0.1838198	1
Cytidine	1220.247	0.7746057	1
Deoxycarnitine	598.3344	0.03979441	1
D-Pantothenic_Acid	1069.784	0.8022101	1
D-Ribose-5-Phosphate	947.4078	0.4893162	1
Fumarate	1122.948	0.9515359	1
Glycerol	587.3005	0.03539137	1
Glycine	1477.036	0.2191074	1
Guanosine	1300.494	0.5653745	1
Homocysteine	1223.256	0.766373	1
Inosine	1243.318	0.7121377	1
L-Arginine	979.5065	0.5653745	1
L-Asparagine	996.5589	0.6077597	1
L-Carnitine	1030.664	0.6961089	1
L-Cystine	1496.095	0.1929174	1
L-Eucine	931.3584	0.453272	1
L-Glutamine	1229.275	0.7499802	1
L-Histidine	1685.678	0.03814706	1
L-Isoleucine	1050.725	0.7499802	1
L-Kynurenine	884.2135	0.3557579	1
L-Methionine	1071.79	0.8077591	1
L-Phenylalanine	709.6768	0.1110978	1
L-Proline	1255.355	0.6802026	1
L-Serine	1090.849	0.8608784	1
L-Threonine	1480.046	0.2148256	1
L-Tyrosine	1270.401	0.6410103	1
Myristic_acid	1745.863	0.01919564	1
Methyl-L-Histidine	1067.778	0.7966701	1
N-Acetyl-DL-Methionine	1224.259	0.7636341	1
N-Acetyl-DL-Serine	1321.558	0.5148828	1
N-Acetylglycine	846.0963	0.2865936	1
N-Acetyl-L-Aspartic_Acid	1468.009	0.2322848	1
N-Acetylputrescine	1481.049	0.2134106	1
Acetyllcarnitine	547.1771	0.02247344	1
Pipecolate	1482.052	0.2120018	1
Spermidine	1304.506	0.5555929	1
Succinate	888.2258	0.3635512	1
Transaconitate	1051.729	0.7527054	1
Uracil	1108.904	0.9117391	1
Urocanate	1222.253	0.7691146	1

```r
which(jia_met_global_spearman_summary$`Adjusted p-value` < .05)
```

```
integer(0)
```

No correlations between metabolite levels and physician global assessment are significant
