# Supplementary material for: Serine, N-acetylaspartate differentiate adolescents with juvenile idiopathic arthritis compared with healthy controls: a metabolomics cross-sectional study
Source: Pediatr Rheumatol Online J. 2022 Feb 10;20:12. doi: 10.1186/s12969-022-00672-z (PMC8832851; doi:10.1186/s12969-022-00672-z)
Supplement: Supplementary file 4 — Additional file 4: Supplementary Table 1. Reported Intake of One or More Medications or Supplements within Each Category within 7 Days Prior to Study Visit. Supplementary Table 2. Overview of Literature about the Role of Serine Metabolism in Arthritis and Related Conditions (plasma serine or serum serine or circulating serine or L-serine) AND (arthritis) AND (metabolome or metabolomics or metabolite). Supplementary Table 3. Overview of Brain Tissue N-Acetylaspartate Literature about Arthritis and Related Diseases. [file 12969_2022_672_MOESM4_ESM.docx]

Supplementary Table 1. Reported Intake of One or More Medications or Supplements within Each Category within 7 Days Prior to Study Visit

| **Category** | **Control** | **JIA** | **Total** |
| --- | --- | --- | --- |
| NSAIDS (Aleve, Ibuprofen, Excedrin, Celebrex); or Tylenol | 3 | 8 | 11 |
| Corticosteroids (prednisolone, solu-medrol) | 0 | 3 | 3 |
| Nonbiologic DMARDS (Xeljanz, leflunomide, methotrexate) | 0 | 8 | 8 |
| Biologic DMARDS (Orencia, Humira, Abatacept, Simponi, Actemra, Enbrel) | 0 | 12 | 12 |
| Allergy (Claritin, Zyrtec, Singulair, Xyzal, Benadryl); Prilosec | 4 | 3 | 7 |
| Antibiotics or Antivirals (Acyclovir, Amoxicillin, Doxycycline) | 3 | 0 | 3 |
| Vitamins, Minerals, and Probiotics (Multi-vitamin, Vit B12, Folic Acid, VD3, Magnesium, Calcium, Fish Oil, Probiotics) | 3 | 9 | 12 |
| Hormones (Focalin, Zoloft, Fluoxetine, Synthroid, Melatonin, Cyclafem) | 4 | 2 | 6 |

Note: NSAID, nonsteroidal anti-inflammatory medication; DMARD, disease-modifying antirheumatic drug.

Supplementary Table 2. Overview of Literature about the Role of Serine Metabolism in Arthritis and Related Conditions (plasma serine or serum serine or circulating serine or L-serine) AND (arthritis) AND (metabolome or metabolomics or metabolite)

| **Author (Year)** | **Disease State** | **Comparison Group** | **Results** | **Key Findings** |
| --- | --- | --- | --- | --- |
| Alonso et al. (2016)^1^ | Immune mediated inflammatory diseases* | Healthy Controls | Glycine-serine metabolism pathway was enriched in the urine samples of immune mediated inflammatory diseases | Glycine-serine pathway metabolites connect the two other pathways that were overrepresented in their samples (the citric acid cycle and phenylalanine pathways) and are associated with anti-inflammatory processes |
| Guo et al. (2021)^2^ | Rheumatoid arthritis, acupuncture intervention group | Rheumatoid arthritis, medication intervention group; healthy control group | Metabolites in the glycine-serine metabolism pathway were downregulated in acupuncture and medication groups compared with controls | The glycine-serine metabolic pathway may be involved in the resolution of symptoms achieved via medications or acupuncture |
| Li et al. (2020)^3^ | Rheumatoid arthritis (review paper) | Healthy controls | Serine was described in 3 studies; serine levels were increased in one study and decreased in two others; overall amino acid trend in rheumatoid arthritis decreased relative to controls | Protein may be degraded into amino acids in response to energy expenditure, inflammation and autoimmunity responses |
| Mickiewicz et al. (2015)^4^ | Idealized surgical repair of knee osteoarthritis (ovine models) | Sham surgical procedure; non-surgical controls (all groups also used the unaffected knee for control) | Serine increased in synovial fluid of surgical model knee joints compared with sham surgical controls | Serine in the synovial fluid may be an early post-injury degenerative disease biomarker; may supply the extra energy needed for collagen and tissue repair |
| Narasimhan et al. (2018)^5^ | Rheumatoid arthritis | Compared serum metabolites with synovial marker gene expression, used ^1^H-nuclear magnetic resonance | Glycine-serine metabolism associated with other pathways that suggest that lymphoid cells use these pathways after activation in the synovial fluid | Serine is important to cell growth and proliferation, may be involved in repair of injured or inflamed synovial tissue |
| Southan et al. (2020)^6^ | Mechanical injury of the hip joint (murine and porcine models) | Integrated omics analysis of murine tissue; cross-analysis of murine and porcine hip joint cartilage | Integrative analysis of transcriptomics and metabolomics finds enriched amino acid metabolism pathways | Glycine-serine pathway and amino acid biosynthesis are enriched in post-injury hip cartilage |
| Zhou et al. (2020)^7^ | Ankylosing spondylitis; rheumatoid arthritis | Healthy controls | Serine significantly decreased in both ankylosing spondylitis and rheumatoid arthritis groups compared with controls; serine correlated with disease activity in ankylosing spondylitis but not rheumatoid arthritis | Glycine-serine pathway was significantly enriched in both ankylosing spondylitis and rheumatoid arthritis, suggesting that both disease states partly share the same metabolic dysregulation |

*Immune-mediated inflammatory diseases include rheumatoid arthritis, psoriasis, psoriatic arthritis, systemic lupus erythematosus, Crohn’s disease, and ulcerative colitis.

1. Alonso A, Julià A, Vinaixa M, Domènech E, Fernández-Nebro A, Cañete JD, et al. Urine metabolome profiling of immune-mediated inflammatory diseases. BMC Med. 2016;14(1):133. https://doi.org/10.1186/s12916-016-0681-8
2. Guo YG, Sun GW, Yang L, Li C, Yang J. [Differential metabolites and metabolic pathways involving acupuncture-induced improvement of rheumatoid arthritis patients based on gas chromatography-mass spectrometry]. Zhen Ci Yan Jiu. 2021;46(2):145-51. Chinese. https://doi.org/10.13702/j.1000-0607.200181
3. Li C, Chen B, Fang Z, Leng YF, Wang DW, Chen FQ, et al. Metabolomics in the development and progression of rheumatoid arthritis: A systematic review. Joint Bone Spine. 2020;87(5):425-30. https://doi.org/10.1016/j.jbspin.2020.05.005
4. Mickiewicz B, Heard BJ, Chau JK, Chung M, Hart DA, Shrive NG, Frank CB, Vogel HJ. Metabolic profiling of synovial fluid in a unilateral ovine model of anterior cruciate ligament reconstruction of the knee suggests biomarkers for early osteoarthritis. J Orthop Res. 2015;33(1):71-7. https://doi.org/10.1002/jor.22743
5. Narasimhan R, Coras R, Rosenthal SB, Sweeney SR, Lodi A, Tiziani S, et al. Serum metabolomic profiling predicts synovial gene expression in rheumatoid arthritis. Arthritis Res Ther. 2018;20(1):164. https://doi.org/10.1186/s13075-018-1655-3
6. Southan J, McHugh E, Walker H, Ismail HM. Metabolic signature of articular cartilage following mechanical injury: an integrated transcriptomics and metabolomics analysis. Front Mol Biosci. 2020;7:592905. https://doi.org/10.3389/fmolb.2020.592905
7. Zhou Y, Zhang X, Chen R, Han S, Liu Y, Liu X, et al. Serum amino acid metabolic profiles of ankylosing spondylitis by targeted metabolomics analysis. Clin Rheumatol. 2020;39(8):2325-36. https://doi.org/10.1007/s10067-020-04974-z

Supplementary Table 3. Overview of Brain Tissue *N*-Acetylaspartate Literature about Arthritis and Related Diseases

keywords: (n-acetyl-aspartate OR n-acetyl-aspartic acid) AND (arthritis)

| **Author (Year)** | **Disease State** | **Comparison Group** | **Results** | **Key Findings** |
| --- | --- | --- | --- | --- |
| Alkan et al. (2004)^1^ | Children post-RF without neuropsychiatric complications | Post-RF with only history of SC; post-RF with SC and secondary obsessive-compulsive tic disorder; healthy controls | NAA/Cr ratio was lowest in post-RF with obsessive-compulsive tic disorder compared to other RF groups and controls  No difference in NAA/Cr ratio between the other groups (SC group only, RF without neuropsychiatric complications) | Decreased NAA post-RF appears to be associated with neuropsychiatric symptom severity. It was reduced only in the group with obsessive-compulsive tic disorder. NAA levels of patients who fully recovered from RF were no different from levels in the healthy control group. |
| Brooks et al. (2010)^2^ | Neuropsychiatric SLE | Healthy controls, post-mortem brains of SLE patients measured pre-mortem | Decreased NAA in SLE vs. controls  Lower NAA levels significantly correlated with decreased neuronal-axonal density in post-mortem SLE brain tissue | Low NAA levels in the brain are correlated with serious underlying histologic brain injury in patients with fatal neuropsychiatric SLE. |
| Emmer et al. (2009)^3^ | RA (study also mentioned briefly in entry for systematic review by Frittoli et al. (2020)^6^ | Healthy controls | Decreased ration of NAA/choline in active RA compared with controls and inactive RA | Systemic inflammation is associated with metabolic changes in the brain for patients with RA |
| Fayed et al. (2010)^4^ | FM | Healthy controls | Decreased NAA in left hippocampus, a region associated with pain and depression, in patients with FM compared with controls | Findings suggest neuronal or axonal metabolic dysfunction, or a combination of these processes, as mechanism of pain, depression, and FM symptoms |
| Foerster et al. (2015)^5^ | FM, pre/post motor cortex transcranial direct current stimulation therapy | Crossover design | NAA increased significantly from baseline to post sham transcranial direct current stimulation therapy | NAA is involved in the pathogenies of FM and modulation of pain |
| Frittoli et al. (2020)^6^ | Rheumatic autoimmune diseases (Review paper; 25 studies measured NAA)   - SLE (21) - Sjögren (1) - RA^3^ (1) - SSc (2) - Behçet´s disease (1) | Varied by study | 14 studies found decrease in NAA or NAA/Cr ratios in comparison with healthy controls for SLE, pSS, SSc, RA; observed in different white matter and grey matter brain regions  Lower NAA/Cr ratios associated with increased disease activity and neuropsychiatric symptoms for SLE; headache, mood disorders, and cerebrovascular reactivity in Sjögren’s Syndrome.  Lower NAA/Cho ratio associated with higher disease activity in RA. | The presence of abnormalities in patients without overt central nervous system manifestations suggests that systemic inflammation, atherosclerosis or abnormal vascular reactivity may be associated with subclinical central nervous system manifestations.  Neurometabolites may be useful biomarkers for the evaluation of neuropsychiatric manifestations in patients with rheumatic autoimmune diseases.  Metabolic alterations may occur without clinical manifestations. |
| Lutz et al. (2013)^7^ | Adjuvant arthritis in rats | Naïve rats and rats with autoimmune encephalomyelitis | Decreased NAA in adjuvant arthritis compared with controls; decrease in NAA matched that in autoimmune encephalomyelitis | Brain NAA levels may suggest either cerebral (autoimmune encephalomyelitis) or extra-cerebral (adjuvant arthritis) related inflammation.  Questions whether disrupted brain metabolites provoked by adjuvant arthritis are pain and stress mediated, or due to systemic proinflammatory molecules |
| Weerasekera et al. (2021)^8^ | Knee osteoarthritis pre- and post TKA patients | Healthy controls | Lower presurgical NAA levels in TKA patients vs controls  After surgery, NAA levels increased in TKA patients, normalized to the level of healthy controls | NAA is associated with neuroinflammation in knee osteoarthritis pain  Reversible mechanism suggests mitochondrial dysfunction as opposed to permanent nerve damage |

Note: RF = rheumatic fever; SC = Sydenham chorea; NAA = *N*-acetylaspartate; Cr = creatinine; SLE = systemic lupus erythematosus; RA = rheumatoid arthritis; FM = fibromyalgia; Sjögren = Sjögren’s syndrome; SSc = systemic sclerosis; pSS = psoriasis; Cho = choline; TKA = total knee arthroplasty; RF = rheumatic fever.

Supplementary References:

1. Alkan A, Kutlu R, Kocak G, Sigirci A, Emul M, Dogan S, et al. Brain MR spectroscopy in children with a history of rheumatic fever with a special emphasis on neuropsychiatric complications. Eur J Radiol. 2004;49(3):224-8. https://doi.org/10.1016/S0720-048X(03)00177-3

2. Brooks WM, Sibbitt WL Jr, Kornfeld M, Jung RE, Bankhurst AD, Roldan CA. The histopathologic associates of neurometabolite abnormalities in fatal neuropsychiatric systemic lupus erythematosus. Arthritis Rheum. 2010;62(7):2055-63. https://doi.org/10.1002/art.27458.

3. Emmer BJ, van der Bijl AE, Huizinga TW, Breedveld FC, Steens SC, Th Bosma GP, et al. Brain involvement in rheumatoid arthritis: a magnetic resonance spectroscopy study. Arthritis Rheum. 2009;60(11):3190-5. https://doi.org/10.1002/art.24932

4. Fayed N, Garcia-Campayo J, Magallón R, Andrés-Bergareche H, Luciano JV, Andres E, Beltrán J. Localized ^1^H-NMR spectroscopy in patients with fibromyalgia: a controlled study of changes in cerebral glutamate/glutamine, inositol, choline, and *N*-acetylaspartate. Arthritis Res Ther. 2010;12(4):R134. https://doi.org/10.1186/ar3072

5. Foerster BR, Nascimento TD, DeBoer M, Bender MA, Rice IC, Truong DQ, et al. Excitatory and inhibitory brain metabolites as targets of motor cortex transcranial direct current stimulation therapy and predictors of its efficacy in fibromyalgia. Arthritis Rheumatol. 2015;67(2):576-81. https://doi.org/10.1002/art.38945

6. Frittoli RB, Pereira DR, Rittner L, Appenzeller S. Proton magnetic resonance spectroscopy (^1^H-MRS) in rheumatic autoimmune diseases: a systematic review. Lupus. 2020;29(14):1873-1884. https://doi.org/10.1177/0961203320961466

7. Lutz NW, Fernandez C, Pellissier JF, Cozzone PJ, Béraud E. Cerebral biochemical pathways in experimental autoimmune encephalomyelitis and adjuvant arthritis: a comparative metabolomic study. PLoS One. 2013;8(2):e56101. https://doi.org/10.1371/journal.pone.0056101

8. Weerasekera A, Morrissey E, Kim M, Saha A, Lin Y, Alshelh Z, et al. Thalamic neurometabolite alterations in patients with knee osteoarthritis before and after total knee replacement. Pain. 2021;162(7):2014-23. https://doi.org/10.1097/j.pain.0000000000002198
